# Improvement of obesity-induced fatty liver disease by intermittent hypoxia exposure in a murine model

**DOI:** 10.3389/fphar.2023.1097641

**Published:** 2023-02-15

**Authors:** Liya Chen, Yao Wang, Weikun Zheng, Hu Zhang, Yan Sun, Yiping Chen, Qi Liu

**Affiliations:** ^1^ Department of Pediatric Infectious Disease, Wenzhou, China; ^2^ The Second Affiliated Hospital & Yuying Children’s Hospital of Wenzhou Medical University, Wenzhou, China; ^3^ Department of Pediatric Hematology Disease, Wenzhou, China

**Keywords:** non-alcoholic fatty liver disease, intermittent hypoxia, inflammation, apoptosis, metabolism

## Abstract

**Background:** The high prevalence of non-alcoholic fatty liver disease (NAFLD) in the world raises an important concern for human health. The western diet containing high fat and fructose is the risk factor for NAFLD development. Intermittent hypoxia (IH), known as the basis of obstructive sleep apnea (OSA), normally is correlated with impaired liver function. However, the role of IH in liver injury prevention has been revealed by many other studies based on the different IH paradigms. The current study, therefore, tests the impact of IH on the liver of high-fat and high-fructose diet (HFHFD) fed mice.

**Material and Method:** Mice were exposed to IH (2 min cycle, FiO_2_ 8% for 20 s, FiO_2_ 20.9% for 100 s; 12 h/day) or intermittent air (FiO_2_ 20.9%) for 15 weeks, with normal diet (ND) or high-fat and high-fructose diet (HFHFD). Indices of liver injury and metabolism were measured.

**Results:** IH causes no overt liver injury in mice fed an ND. However, HFHFD-induced lipid accumulation, lipid peroxidation, neutrophil infiltration, and apoptotic process were significantly attenuated by IH exposure. Importantly, IH exposure altered bile acids composition and shifted the hepatic bile acids towards FXR agonism, which was involved in the protection of IH against HFHFD.

**Conclusion:** These results support that the IH pattern in our model prevents liver injury from HFHFD in experimental NAFLD.

## Introduction

NAFLD is a spectrum of liver disease that ranges from simple steatosis, non-alcoholic steatohepatitis to fibrosis, and cirrhosis, eventually leading to liver cancer. It is reported that over 75% of individuals having NAFLD and it becomes the most prevalent liver disease in the world (Younossi et al., 2011). There are multiple risk factors that related to NAFLD development. The ingestion of high-calorie food is strongly associated with fatty liver disease. Emerging evidence showed that overconsumption of fat or fructose resulted in energy metabolic disruption, impaired mitochondrial dysfunction and autophagy dysregulation, leading to liver damage (Chen et al., 2019). Since western fast food is escalating in prevalence globally, obesity-associated NAFLD has become one of the most important public health problems.

Current studies suggest that obesity is the common risk factor for sleep-disordered breathing conditions (Murphy et al., 2017). Obstructive sleep apnea (OSA) is characterized by recurrent episodes of upper airway obstruction with recurrent cycles of desaturation that leads to intermittent hypoxia (IH) and fragmentation of sleep associated with daytime fatigue and sleepiness (Mesarwi et al., 2019). It is reported that 60%–70% of the obese population has OSA, and the risk is approximately 12–30 times greater than that of subjects with a normal weight (Young et al., 2002; Shah and Roux, 2009). As the hallmark of OSA, IH plays a role in oxidative stress, inflammation, and insulin resistance (Sforza and Roche, 2016; Murphy et al., 2017), suggesting that OSA is a potential risk factor for liver injury. OSA was found to be associated with elevated alanine aminotransferase levels and progressive liver diseases (Schwenger et al., 2020; Bahr and Simon, 2021). Jian Zhou et al. verified that IH increased western diet-induced steatohepatitis in mice (Zhou et al., 2021). The data above demonstrated that IH exacerbated liver function caused by obesity. However, some studies performed the opposite result that IH is also beneficial to physiological performance based on the condition of lacking oxygen. IH condition was considered as an adaptive process increasing cellular tolerance to other severer conditions such as ethanol withdrawal stress or ischemia (Neckář et al., 2004; Ju et al., 2012). These findings suggested that IH exposure might be a therapeutic technique for liver injury caused by harmful stresses such as dietary fat. Indeed, Hideyuki Maeda et al. reported that IH limited hepatic pathogenesis in rats fed with a high-fat diet by enhancing antioxidative stress (Maeda and Yoshida, 2016). Wojciech Trzepizur et al. indicated IH protection in high-fat diet-induced liver injury was mediated by restoring mitochondrial dysfunction and decreasing triglyceride accumulation (Trzepizur et al., 2015). These data showed completely different outcomes that IH played a role in protecting liver injury caused by dietary fat. Nevertheless, the detailed mechanisms by which IH ameliorates NAFLD remain unclear.

We suspected that IH showing controversial effects in obese rodents was due to the diversity and duration of IH patterns applied. Herein, we attempted to establish a model of NAFLD in.

C57BL6 mice with extra fat and fructose ingestion, explored the impact of IH exposure on liver function as well as investigated the mechanisms involved, such as energy and bile acids metabolism, oxidative stress, inflammation, and apoptosis.

## Materials and methods

### Animals and treatment

**FIGURE 1:**
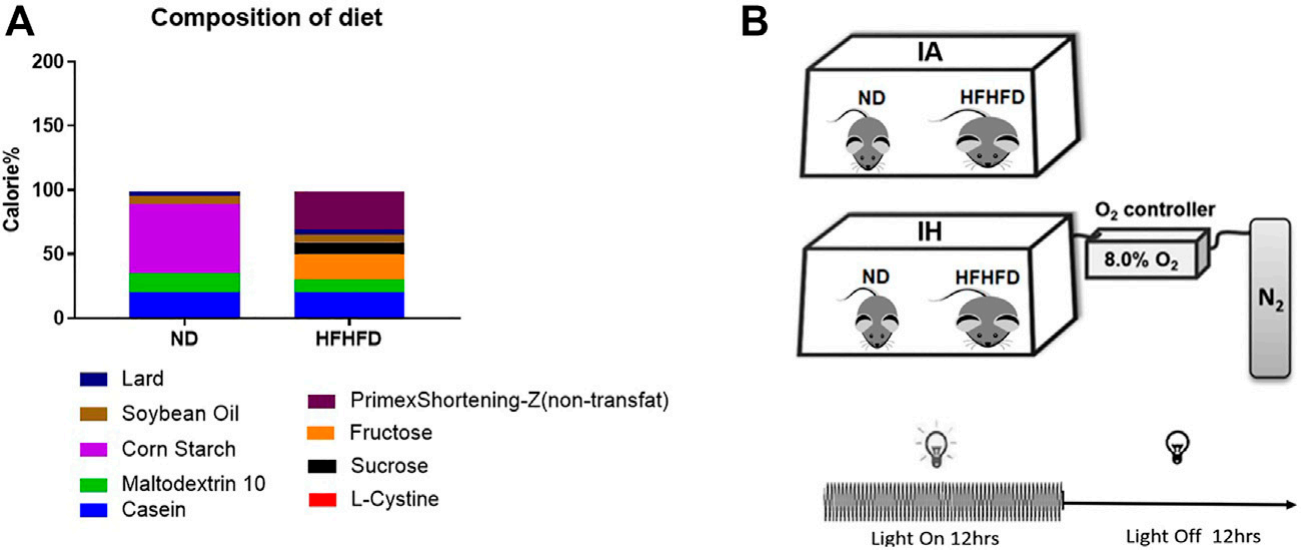
Diet and treatment. **(A)** Food composition and calorie ratio of the individual components were provided in ND and HFHFD. **(B)** The IH chamber was monitored with an automated gas controller and designed for mimicking different levels of hypoxia exposure by regulating the ratio of the gaseous mixture. Mice were exposed to IH (20.9% O_2_/8% O_2_ FiO_2_) and normoxia during the 12-h light phase, respectively. The mice were divided into four groups: ND + IA, ND + IH, HFHFD + IA, and HFHFD + IH.

Six-week-old C57BL/6 wild-type male mice were obtained from Beijing Vital River Laboratory Animal Technology Co., Ltd., (Beijing, China). The mice were housed (five per cage) in a conventional animal room and subjected to a 12:12 h light/dark cycle in low-stress conditions with free access to food and water *ad libitum*. The mice of the control group were fed a normal diet (ND) containing 10% kcal fat (cat. no. D12450J; Research Diet, New Brunswick, NJ, United States) and regular tap water. The western diet group mice were fed with a liquid high-fat: high-fructose diet (HFHFD) containing 40% kcal fat and 20% kcal fructose (made up with cultured media) (cat. no. D09100308L; Research Diet, New Brunswick, NJ, United States) The food composition and calories were shown in Figure 1A and supplemental material. Additionally, the mice were exposed to IH during their sleeping hours or intermittent room air (IA) as control conditions for 15 weeks, and started treatment with the diet concurrently.

The IH chamber was monitored with an automated gas controller and designed for mimicking different levels of hypoxia exposure by regulating the ratio of the gaseous mixture. As described in our previous work, the IH paradigm was consisted of repeated cycles of 20.9% O_2_/8% O2 FiO_2_ (30 episodes per hour) with 20 s at the nadir FiO_2_ during the 12-h light phase (Liu et al., 2020) (Figure 1B). The mice were divided into four groups: ND + IA, ND + IH, HFHFD + IA, and HFHFD + IH. All the animal procedures were approved by the Animal Experimental Ethical Inspection of the Laboratory Animal Centre of Wenzhou Medical University prior to the start of the study.

### Sample collection

The body weight and food consumption of animals were monitored weekly. In the 15th week, the mice were anesthetized with ketamine/xylazine (100/15 mg/kg, i.p.) and sacrificed. Blood was collected from the vena cava by cardiac puncture prior to exsanguination, centrifuged to separate serum, and then stored at −80°C. Epididymal fat pads and liver were harvested and weighed. Portions of the liver, intestine, and adipose tissue were frozen in liquid nitrogen or fixed in 10% neutral buffered formalin for further analysis.

### Biochemical analyses, histology

The day before sacrificing, the mice were fasted overnight and underwent a glucose tolerance test (GTT) and insulin tolerance test (ITT). Glucose solution (2 g/kg) and insulin (0.75 U/kg) were administered by intraperitoneal injection. Tail blood was collected at 0, 15, 30, 60 and 120 min after glucose administration, and glucose concentrations were measured using a glucometer and test strips (ACCU-CHEK Performa; Roche, United States). Serum alanine aminotransferase (ALT) and aspartate aminotransferase (AST) levels were determined by using standard kits purchased from Cusabio (Wuhan, China). Hepatic, adipose or serum lipids including triglyceride (TG), free fatty acid (FFA) and cholesterol, were determined by commercial lipids assay kits (abcam, Cambridge, MA, United States) according to the manufacturer’s protocols. Four-hydroxynonenal (4-HNE) level in the liver was measured by colorimetric assay using an ELISA kit (Cusabio, China). Formalin-fixed mouse liver and epididymal white adipose tissues were embedded in paraffin and sliced at 4.5 μm. Hematoxylin and eosin (H&E) staining was used for tissue morphology assessment. Pathology was scored in a blinded manner. Scoring ranges were as follows: Degree of steatosis (0–3), lobular inflammation (0–3), hepatocyte ballooning (0–2). In addition, the NAFLD activity score (NAS) was scored according to the non-alcoholic steatohepatitis (NASH) clinical research network scoring system (Kleiner et al., 2005). Hepatic lipids were visualized by Oil Red O (ORO) stain (Servicebio, Wuhan, China). Image Pro plus 6.0 was used for ORO staining quantification. Neutrophil infiltration in the liver was assessed by a naphthol AS-D chloroacetate (CAE) kit (Sigma-Aldrich, United States) according to the manufacturer’s directions. Apoptosis was detected by terminal deoxynucleotidyl transferase–mediated deoxyuridine triphosphate nick-end labeling (TUNEL; EMD Millipore, Billerica, MA). CAE and TUNEL positive cells were counted by Image J software (NIH, United States) and expressed as the ratio of positive cells per 1,000 hepatocytes.

### Immunoblots

Protein was extracted from frozen liver and epididymal white adipose tissues (eWAT). Nuclear protein was prepared from mouse livers as described previously (Alam et al., 2017). Protein concentration was determined using a Pierce BCA Protein Assay kit (Thermo Fisher Scientific, MA). Western blotting was performed as described previously (Liu et al., 2020). Primary antibodies (1:500–1:1,000) against Phospho-HSL, ATGL, PPAR-γ, CPT-1a, Nrf2, BAX, BCL-2, Adiponectin, Cleaved-Caspase3, pro-Caspase3 and CYP7A1 were from Cell Signaling Technology (MA, United States). β-Actin and Lamin B antibody were the reference of total protein and nuclear protein, respectively, and purchased from Abcam (Cambridge, MA, United States). Densitometric analysis was performed using UN-SAN-IT Gel (Silk Scientific, Orem, UT) software.

### Real-time quantitative PCR

Total mRNA was extracted from fresh liver tissue, eWAT and ileum by TRIzol (Invitrogen) reagent according to the manufacturer’s protocol. Total RNA was used for reverse transcription with the cDNA cycle kit (Invitrogen). Primers for the experiment are listed in Table 1. Real-time PCR was performed on the ABI 7300 fast real-time PCR system (Applied Biosystems) using SYBR green PCR Master Mixture (Applied Biosystems). The 2^−△△CT^ method was used to determine fold differences between the target genes and an endogenous reference (GAPDH).

**TABLE 1:** Primer sequences used for real-time PCR analysis.

Gene name	Forward primer 5′–3′	Reverse primer 5′–3′
Srebp-1c	GGA​GCC​ATG​GAT​TGC​ACA​TT	CCT​GTC​TCA​CCC​CCA​GCA​TA
Scd-1	TGT​CTC​GGT​GTG​TGT​CGG​AGT	TGT​ACC​ACT​ACC​TGC​CTG​CAT​G
Chrebp	GAG​TGC​TTG​AGC​CTG​GCT​TAC​A	GCT​CTC​CAG​ATG​GCG​TTG​TTC​A
Fasn	TTG​CTG​GCA​CTA​CAG​AAT​GC	AAC​AGC​CTC​AGA​GCG​ACA​AT
Cd36	TTC​CAG​CCA​ATG​CCT​TTG​C	TGG​AGA​TTA​CTT​TTT​CAG​TGC​AG
Fatp5	CTG​CGG​TAC​TTG​TGT​AAC​GTC​C	TCC​GAA​TGG​GAC​CAA​AGC​GTT​G
SOD	TAA​CGC​GCA​GAT​CAT​GCA​GCT​G	AGG​CTG​AAG​AGC​GAC​CTG​AGT​T
CAT	CTG​TTC​CCT​TCC​CTG​CTG​GAT​A	TCA​CCA​AGG​CAG​CAT​GGA​CAA​C
Cpt-1α	GGC​ATA​AAC​GCA​GAG​CAT​TCC​TG	CAG​TGT​CCA​TCC​TCT​GAG​TAG​C
TNFα	GGT​GCC​TAT​GTC​TCA​GCC​TCT​T	GCC​ATA​GAA​CTG​ATG​AGA​GGG​AG
HIF-1a	CCT​GCA​CTG​AAT​CAA​GAG​GTT​GC	CCA​TCA​GAA​GGA​CTT​GCT​GGC​T
BNIP3	GCT​CCA​AGA​GTT​CTC​ACT​GTG​AC	GTT​TTT​CTC​GCC​AAA​GCT​GTG​GC
MCP-1	GCT​ACA​AGA​GGA​TCA​CCA​GCA​G	GTC​TGG​ACC​CAT​TCC​TTC​TTG​G
CXCL1	TCC​AGA​GCT​TGA​AGG​TGT​TGC​C	AAC​CAA​GGG​AGC​TTC​AGG​GTC​A
IL-1β	CAA​CCA​ACA​AGT​GAT​ATT​CTC​CAT​G	GATCCACACTCTC CAGCTGCA
IL-6	CCG​GAG​AGG​AGA​CTT​CAC​AGA	AGA​ATT​GCC​ATT​GCA​CAA​CTC​TT
ANGPTL2	GCG​ACT​CCT​TTA​CCT​GGC​ACA​A	GTT​GGA​GTG​AGC​ACA​GGC​GTT​A
FXR	TCC​ACA​ACC​AAG​TTT​TGC​AG	TCT​CTG​TTT​GTT​GTA​CGA​ATC​CA
SHP	AAG​GGC​ACG​ATC​CTC​TTC​AA	CTG​TTG​CAG​GTG​TGC​GAT​GT
ASBT	TGG​GTT​TCT​TCC​TGG​CTA​GAC​T	TGT​TCT​GCA​TTC​CAG​TTT​CCA​A

### Bile acids quantification

Quantitative analysis of hepatic bile acids was measured following published methods with modifications (García-Cañaveras et al., 2012; Xie et al., 2013; Xie et al., 2015). Mouse liver homogenate samples or standard solutions were mixed with acetonitrile (1:3, v/v) containing internal standards, then the extractions were centrifuged and the supernatants were combined and dried. The dried residues were resuspended in acetonitrile/methanol (95:5, v/v) and centrifuged at 13,500 *g* at 4°C for 20 min for further solid-phase extraction. The supernatant was transferred to a 96-well plate for LC-MS analysis. A Waters ACQUITY ultra performance LC (UPLC) system equipped with a binary solvent delivery manager and a sample manager (Waters, Milford, MA) was used throughout the study. UPLC-MS raw data obtained with negative mode were analyzed using TargetLynx applications manager version 4.1 (Waters Corp., Milford, MA) to obtain calibration equations and the quantitative concentration of each bile acid in the samples. Total hepatic bile acids were calculated from the sum of individual bile acid species and normalized to tissue weight.

### Statistical analysis

Results were reported as means ± SEM (N = 4–6) and analyzed using the GraphPad Prism version 6 (GraphPad Software Inc., San Diego, CA, United States). Two-way analysis of variance (ANOVA) with Bonferroni *post hoc* test or one-way ANOVA with Tukey’s *post hoc* test or Student’s t-test where it was appropriate. A *p*-value <0.05 was considered to be a significant difference.

## Result

### The metabolic phenotype of mice affected by HFHFD and IH treatment

**FIGURE 2:**
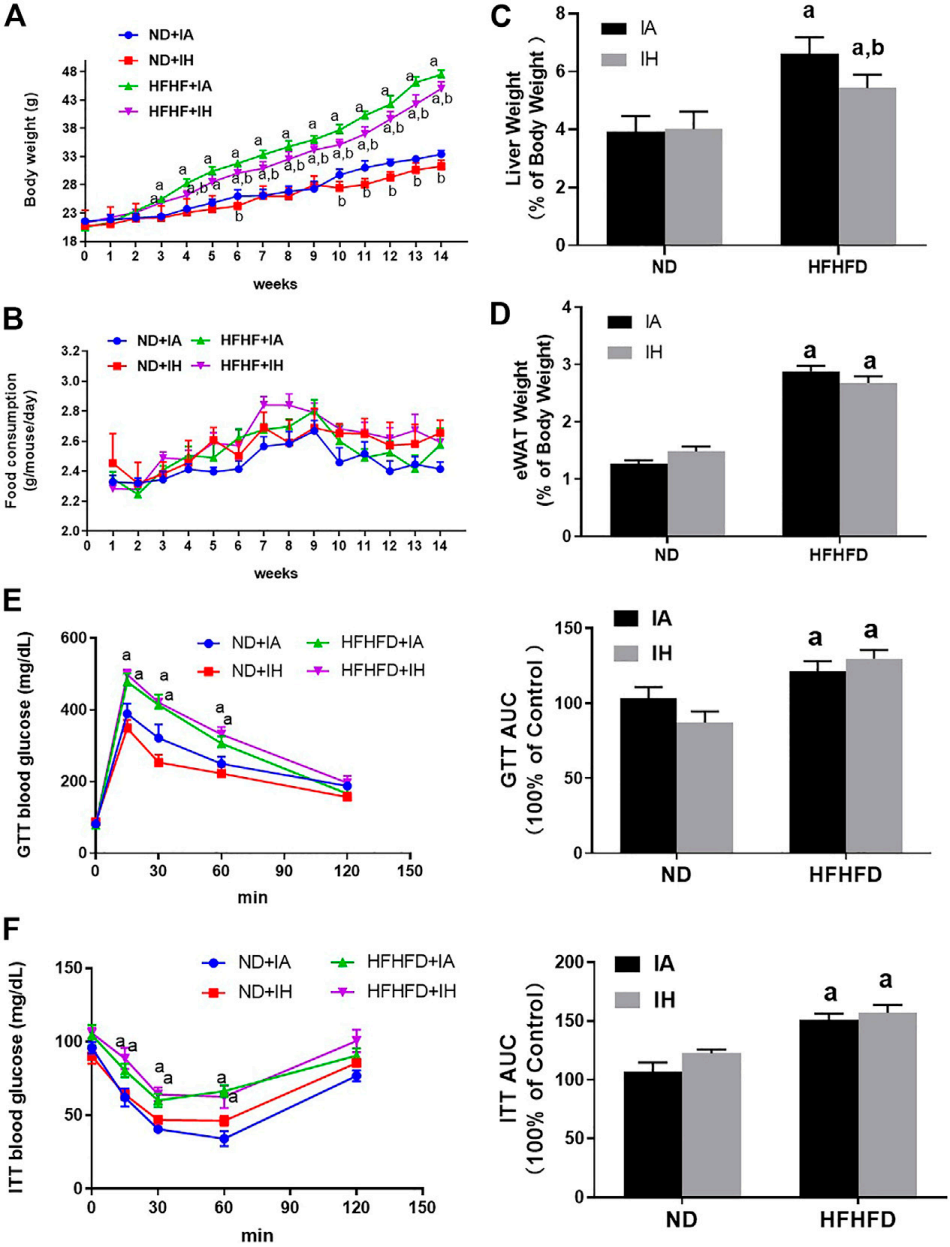
Metabolic phenotype. **(A)** The body weight of mice for all groups was monitored once per week and is depicted for 15°weeks. **(B)** Food consumption was measured twice per week during the 15-week exposure period. **(C,D)** Liver weight to body weight ratios and eWAT weight to body weight ratios were calculated for each group at the 15th week. **(E,F)** Glucose tolerance tests (GTT) and insulin tolerance tests (ITT) were performed and the area under the curve (AUC) was calculated. a, *p* < 0.05 compared to ND control, b, *p* < 0.05 compared to absence of IH. Samples size per group n = 4–6.

During the period of animal modeling, mice body weight and food consumption were monitored weekly. There was no significant difference in food intake observed in all the groups (Figure 2B). Mice of HFHFD showed significant weight gain from the third week by comparison to their control groups, and IH treatment significantly decreased the mice body weight in both ND and HFHFD groups at the 15th week (Figure 2A). HFHFD increased liver and eWAT weight as expressed by the ratio to body weight. IH decreased liver to body weight ratios in the HFHFD feeding mice, however, the parameter for eWAT was not significantly altered by IH exposure (Figures 2C, D). To assess mice glucose metabolism, we determined GTT and ITT at the end of the experimentation period. HFHFD-fed mice showed a decreased glucose tolerance as represented by elevated peak blood glucose levels at the 15th min time point compared to that of ND mice (Figure 2E). Additionally, HFHFD decreased insulin sensitivity as determined by the ITT measurement (Figure 2F). However, no differences in GTT or ITT were found between HFHFD + IA and HFHFD + IH groups.

### IH exposure attenuated liver injury caused by HFHFD

**FIGURE 3:**
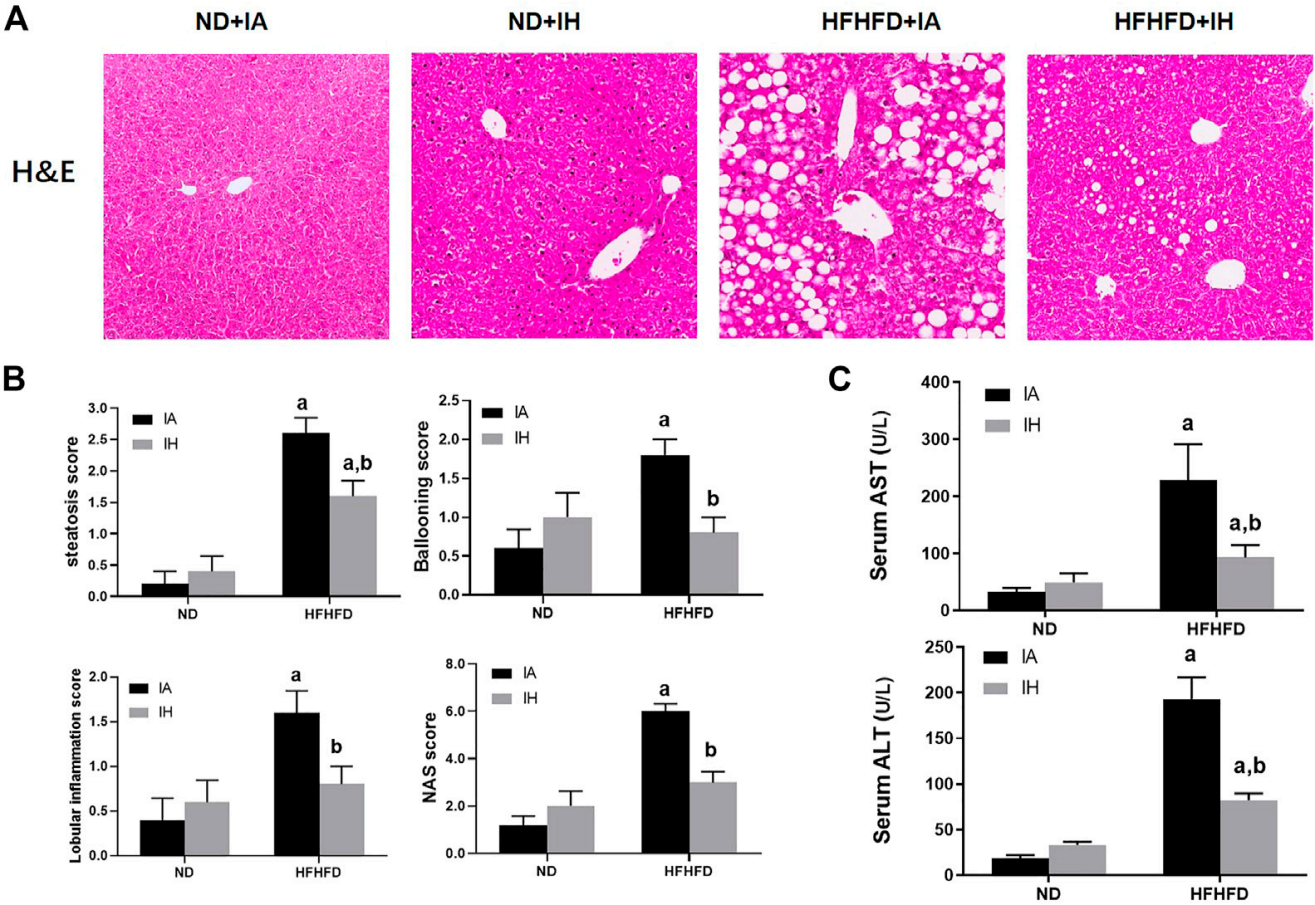
IH decreased liver injury caused by HFHFD. **(A)** Representative photomicrographs of H&E (general morphology, 200x) are shown of mice sacrificed at 15 weeks. **(B)** Scores for steatosis, ballooning, lobular inflammation and NAS for the four experimental groups. **(C)** Serum transaminase (ALT/AST) levels were determined for all the experimental groups at the 15-week time point. a, *p* < 0.05 compared to ND control, b, *p* < 0.05 compared to absence of IH. Samples size per group n = 4–6.

General hepatic morphology was visualized by representative microphotographs of H&E staining. Figure 3B showed that normal histology was observed in the ND group, and IH caused no overt pathological change in the presence of ND. Compared with mice in the ND group, significant pathological findings such as marked steatosis, ballooning hepatocytes, lobular inflammation, and NAS were observed in the HFHFD group. IH exposure alleviated these morphological changes and decreased NAS score in the HFHFD feeding mice. Similarly, elevated transaminases including ALT and AST in HFHFD mice were significantly decreased by IH (Figure 3C). Taken together, these data suggested that IH reduced HFHFD-associated liver injury.

### IH exposure alleviated hepatosteatosis and oxidative stress in the HFHFD

**FIGURE 4:**
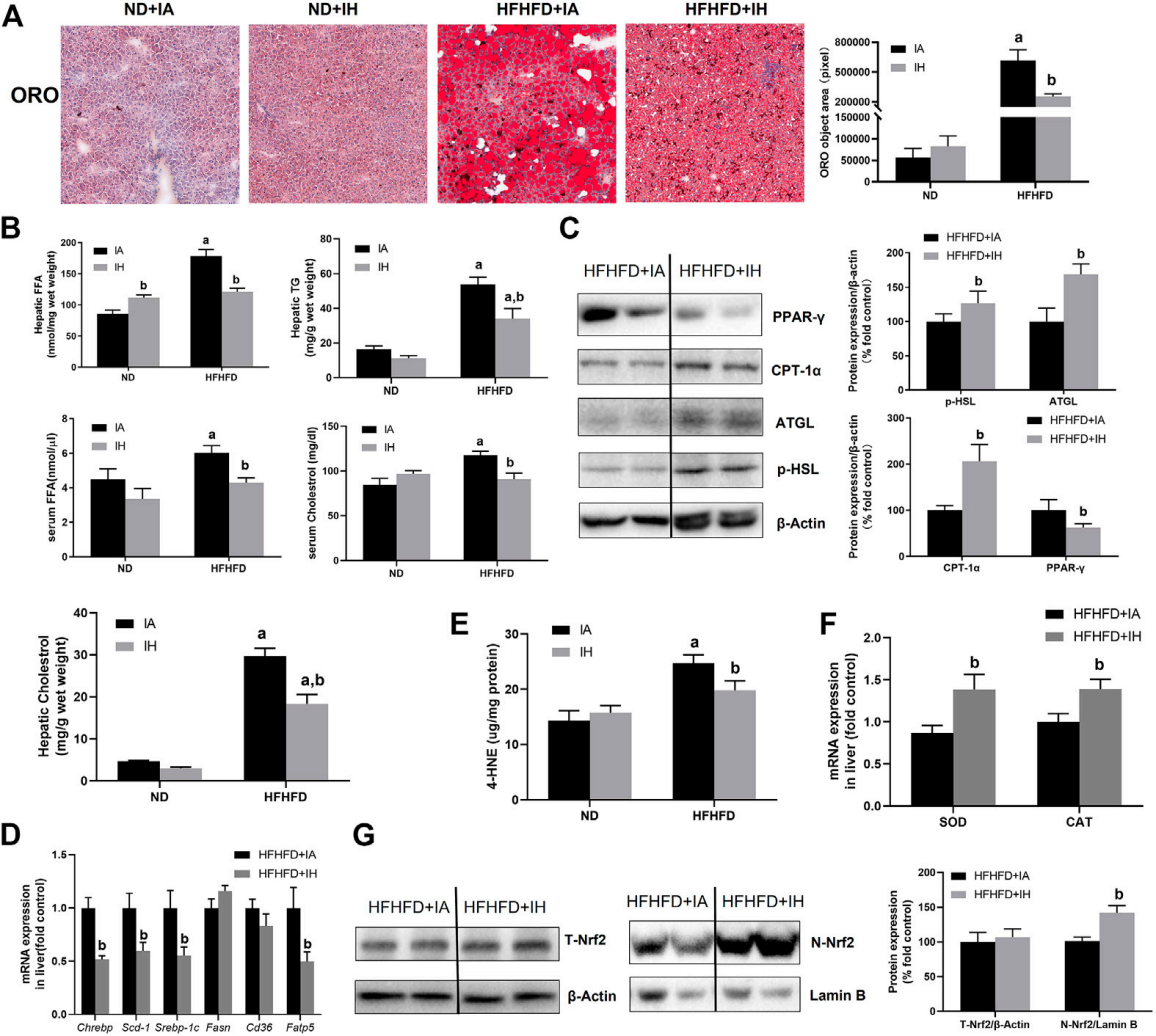
IH decreased lipid accumulation and increased antioxidant genes expression in the liver in HFHFD-fed mice. **(A)** Representative photomicrographs of ORO (neutral lipids, red) staining at ×200 magnification are shown. Right panel: ORO staining quantifications. **(B)** Hepatic or serum cholesterol, TG and FFA content were measured as described in Material and Methods. **(C)** Representative Western blot analyses for lipid metabolism-associated proteins in liver extracts: p-HSL, ATGL, PPAR-γ and CPT-1α. Densitometric analysis for these markers is shown in HFHFD + IA and HFHFD + IH. **(D)** Hepatic mRNA expression of lipogenesis markers Srebp-1c, Chrebp, Scd-1, Fasn, Fatp5 and Cd36 are shown. **(E)** Hepatic 4-HNE levels (ug/mg protein) were measured by ELISA kit in all groups, as described in Material and Methods. **(F)** mRNA expression of antioxidative enzyme SOD and CAT in the liver are shown. **(G)** Representative Western blot analyses for Nrf2 in liver extracts. Densitometric analysis for these markers is shown in HFHFD + IA and HFHFD + IH. a, *p* < 0.05 compared to ND control, b, *p* < 0.05 compared to absence of IH. Samples size per group n = 4–6

Liver sections were stained for ORO to evaluate lipids storage (Figure 4A). HFHFD exacerbated steatosis was alleviated by IH treatment. Quantitative lipids content showed a similar trend to staining. FFA, TG, and cholesterol levels in both liver and serum were increased by HFHFD, while IH significantly decreased these targets (Figure 4B). To investigate the mechanisms of reduced steatosis, we measured hepatic mRNA as well as protein levels of typical enzymes or transcriptional factors involved in lipid metabolism in the group of HFHFD + IA and HFHFD + IH (Figures 4C, D). HSL and ATGL are the key enzymes that catalyze lipolysis (Grabner et al., 2021). Cd36 and Fatp5, are known as the major players that mediate fatty acids uptake (Pohl et al., 2004; Hao et al., 2020). CPT-1α is the rate-limiting enzyme for fatty acid β-oxidation (Schlaepfer and Joshi, 2020). Fasn, Chrebp, Srebp-1c and Scd-1 are the important transcription factors associated with lipogenesis (Yu et al., 2018). PPAR-γ can be activated by Chrebp and participates in lipid synthesis (Witte et al., 2015). Our data showed that IH enhanced the protein expression of p-HSL, AGTL and CPT-1α in the HFHFD group while decreasing PPAR-γ level as determined by western blot. For gene expression levels, IH significantly decreased mRNA expression levels of Fatp5, Chrebp, Srebp-1c and Scd-1 in HFHFD mice, whereas Cd36 and Fasn were not altered by IH (Figures 4C, D). We furtherly measured oxidative stress in the liver. 4-HNE, the main byproduct of lipid peroxidation, was elevated in HFHFD compared to their control mice. IH exposure caused no obvious change in the 4-HNE level of ND mice but reduced it in the mice of HFHFD (Figure 4E). Nrf2, known as the famous antioxidant, was also determined in our work. IH caused no change in total Nrf2 expression in HFHFD liver, but significantly increased nuclear Nrf2 expression level (Figure 4G). Moreover, the well-known Nrf2 downstream target genes, SOD and CAT, were upregulated in the interaction of IH + HFHFD mice (Figure 4F). These data proved that IH exposure protected liver from HFHFD *via* manipulating lipid metabolism and suppressing hepatic oxidative stress.

### IH suppressed lipolysis and promoted lipogenesis in eWAT

**FIGURE 5:**
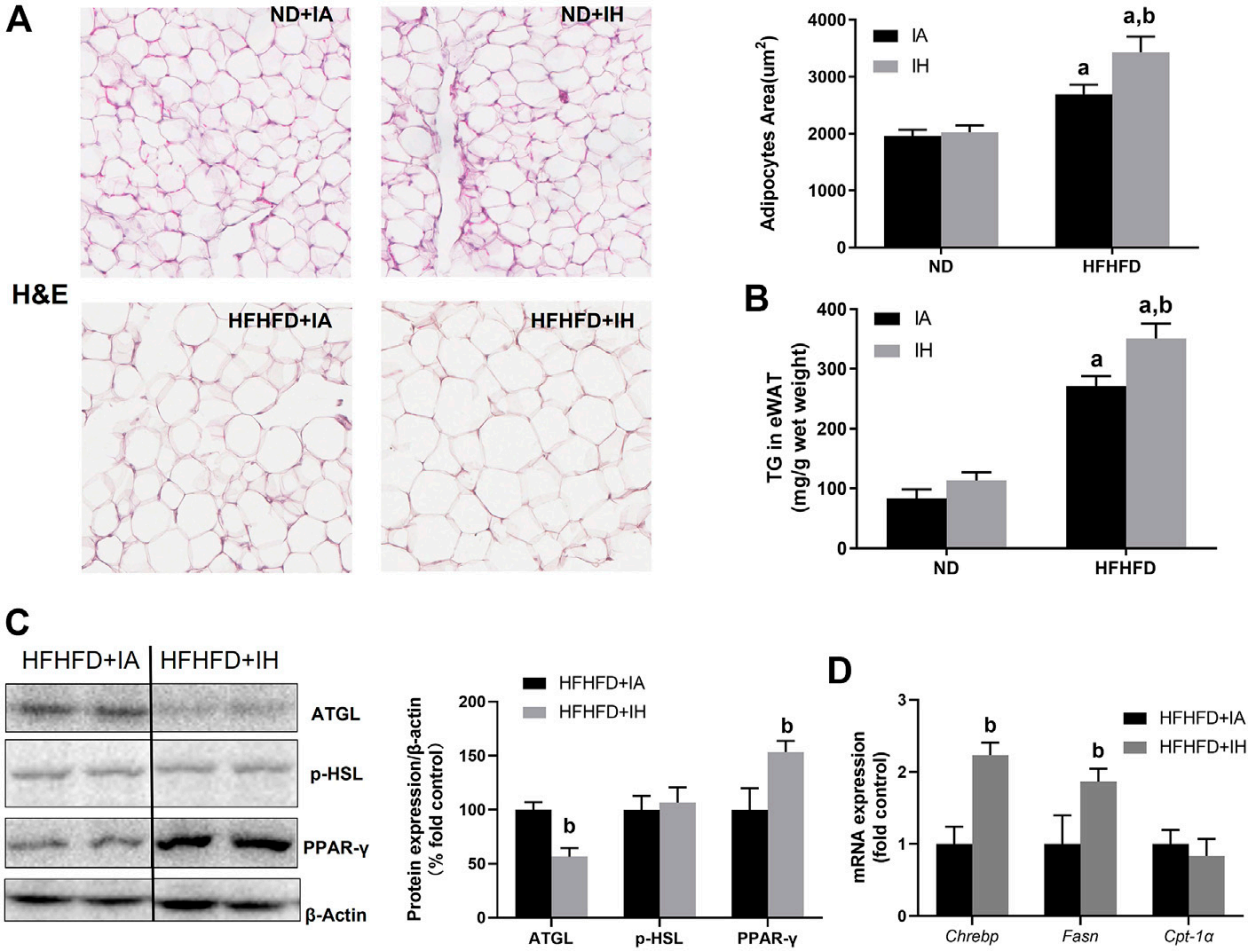
IH increased steatosis in eWAT in HFHFD-fed mice. **(A)** Representative H&E staining of white adipose tissues (eWAT) (×300) and semi quantification of adipocyte size are shown. **(B)** TG concentration of eWAT was measured as described in Material and Methods. **(C)** Representative western blot analyses and densitometric analysis for lipid metabolism-associated proteins in adipose tissue: p-HSL, ATGL and PPAR-γ. **(D)** Hepatic mRNA expression of Chrebp, Fasn, and CPT-α are performed in HFHFD–fed mice. a, *p* < 0.05 compared to ND control, b, *p* < 0.05 compared to absence of IH. Samples size per group n = 4–6.

The finding of decreased serum FFA levels in HFHFD + IH mice suggested that lipid metabolism in adipose tissue was regulated by IH. Therefore, we determined the general pathological feature of eWAT by H&E staining. HFHFD increased lipid storage in adipose tissue and that was enhanced by IH exposure, which was proved by adipocyte size evaluation (Figure 5A) as well as the quantitative TG level in eWAT (Figure 5B). The major enzymes of fat mobilization in adipose tissue were also measured. IH caused a significant reduction of AGTL protein expression but not p-HSL. And the factors involved in lipogenesis including PPAR-γ, Fasn and Chrebp, were increased by IH in the HFHFD mice, as measured by western blot and rt-qPCR (Figures 5C, D). However, CPT-1α, which served as the critical enzyme for FFA oxidation, was not affected by IH (Figure 5D).

### IH decreased neutrophil infiltration and inflammation in the liver of HFHFD

**FIGURE 6:**
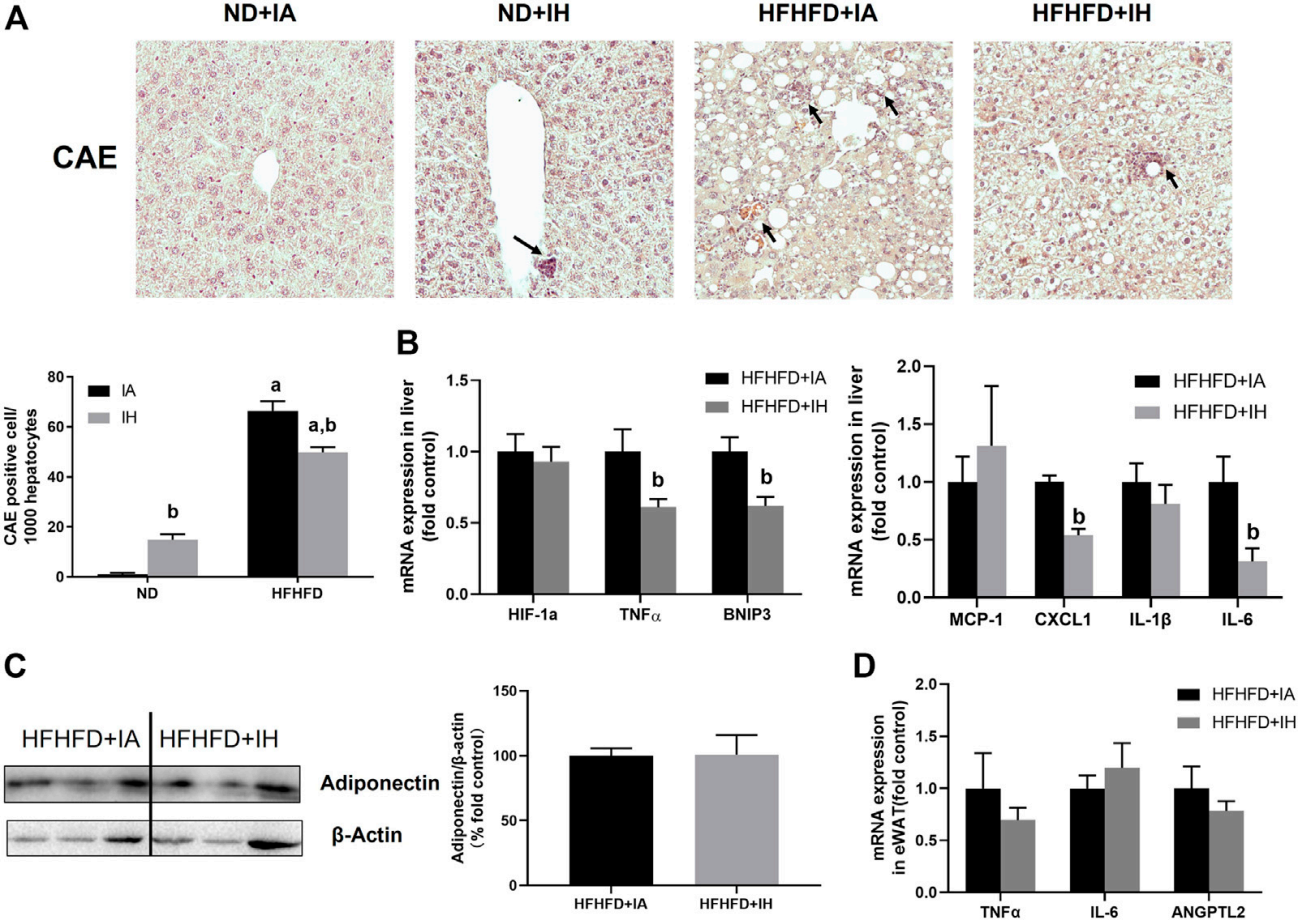
IH reduced inflammation in HFHFD-fed mice. **(A)** Representative photomicrographs of CAE (neutrophils, purple) are shown at ×200 magnification. CAE-positive cells were counted and graphed as positive cells per 1,000 hepatocytes. **(B)** Hepatic expressions of genes related to inflammation are shown. **(C)** Representative western blot analyses and densitometric analysis for adiponectin in adipose tissue are performed. **(D)** mRNA expression of pro-inflammatory adipokines in HFHFD + IA and HFHFD + IH mice are shown. a, *p* < 0.05 compared to ND control, b, *p* < 0.05 compared to absence of IH. Samples size per group n = 4–6.

To evaluate liver inflammation, CAE staining and genes involved in inflammatory modulation were performed. Visible CAE-positive staining was observed in the HFHFD + IA group, which was also reflected in cell counting. IH exposure significantly increased neutrophil recruitments in the absence of HFHFD, while it showed a robust reduction in neutrophil infiltration in the mice of HFHFD (Figure 6A). CXCL1 is the main chemokine that controls neutrophil recruitment and contributes to inflammation. A decreased mRNA expression level of CXCL1 was shown in the HFHFD + IH compared to HFHFD + IA mice. MCP-1 is the key chemokine that regulates the migration and infiltration of monocytes/macrophages. In the HFHFD + IH mice, the expression of MCP-1 was not statistically different from that of HFHFD + IA mice. The pro-inflammatory cytokines including IL-6 and TNFα but not IL-1β were inhibited by IH exposure in mice treated with HFHFD. In addition, IH reduced other inflammatory mediator gene levels such as BNIP3 in the presence of HFHFD, but no significant change in the HIF-1a expression was observed in these groups (Figure 6B). Furthermore, we measured adipokines expression in the eWAT for inflammation assessment. The relative protein level of adiponectin was not affected by IH in the HFHFD mice (Figure 6C). And the mRNA levels of pro-inflammatory adipokines TNFα, IL-6 and ANGPTL2, were not significantly different between HFHFD + IA and HFHFD + IH (Figure 6D).

### IH ameliorated hepatic apoptosis induced by HFHFD

**FIGURE 7:**
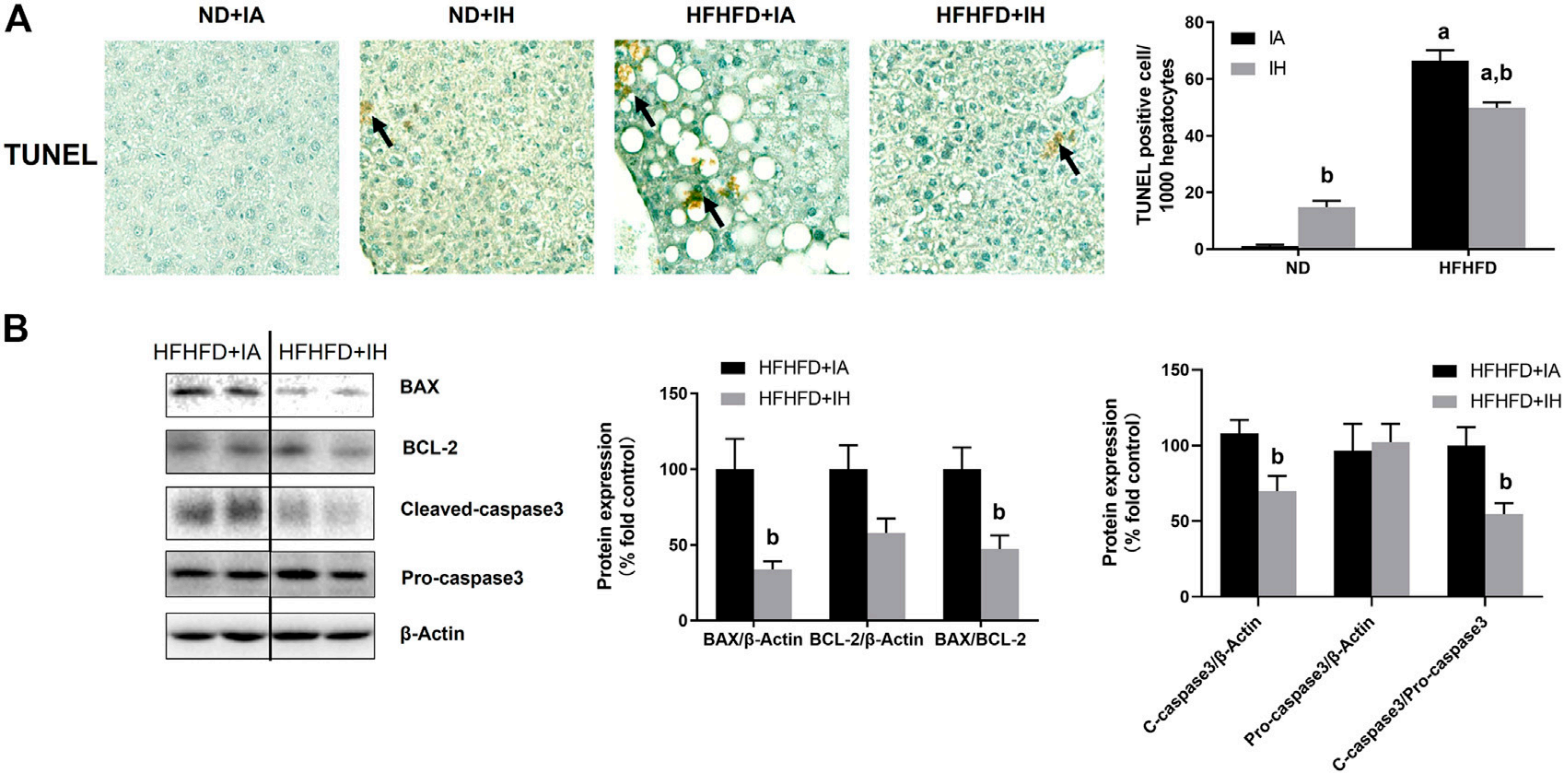
IH alleviated liver apoptosis in HFHFD-fed mice. **(A)** TUNEL staining was performed for the 15-week samples as presented by photomicrographs (magnification ×200). TUNEL-positive cells were quantified and are expressed as TUNEL-positive cells per 1,000 hepatocytes. **(B)** Representative western blots and densitometric analysis for whole liver Caspase-3, BAX, and BCL-2 protein are shown. a, *p* < 0.05 compared to ND control, b, *p* < 0.05 compared to absence of IH. Samples size per group n = 4–6.

For histologic analysis of apoptosis, representative photomicrographs of the TUNEL staining in the liver and positive cell counting were performed in Figure 7A. HFHFD increased TUNEL-positive stained cell counts of hepatocytes and non-parenchymal cells compared to ND. IH caused a severer degree of TUNEL staining in the ND mice but moderated this phenomenon in the HFHFD (Figure 7A). For assessment of cell death pathway, Caspase3, BAX and BCL-2 protein levels were analyzed by western blot. IH decreased caspase3 cleavage and BAX expression in the liver of HFHFD mice, while did no significant change in pro-caspase3 and BCL-2 protein levels. The ratio of cleaved-to pro-caspase3 and the ratio of BAX to BCL-2 were downregulated by IH exposure in the HFHFD (Figure 7B), indicating that IH decreased apoptosis caused by HFHFD.

### IH decreased hepatic bile acids content and altered the composition of the bile acids pool in the HFHFD-fed mice

**FIGURE 8:**
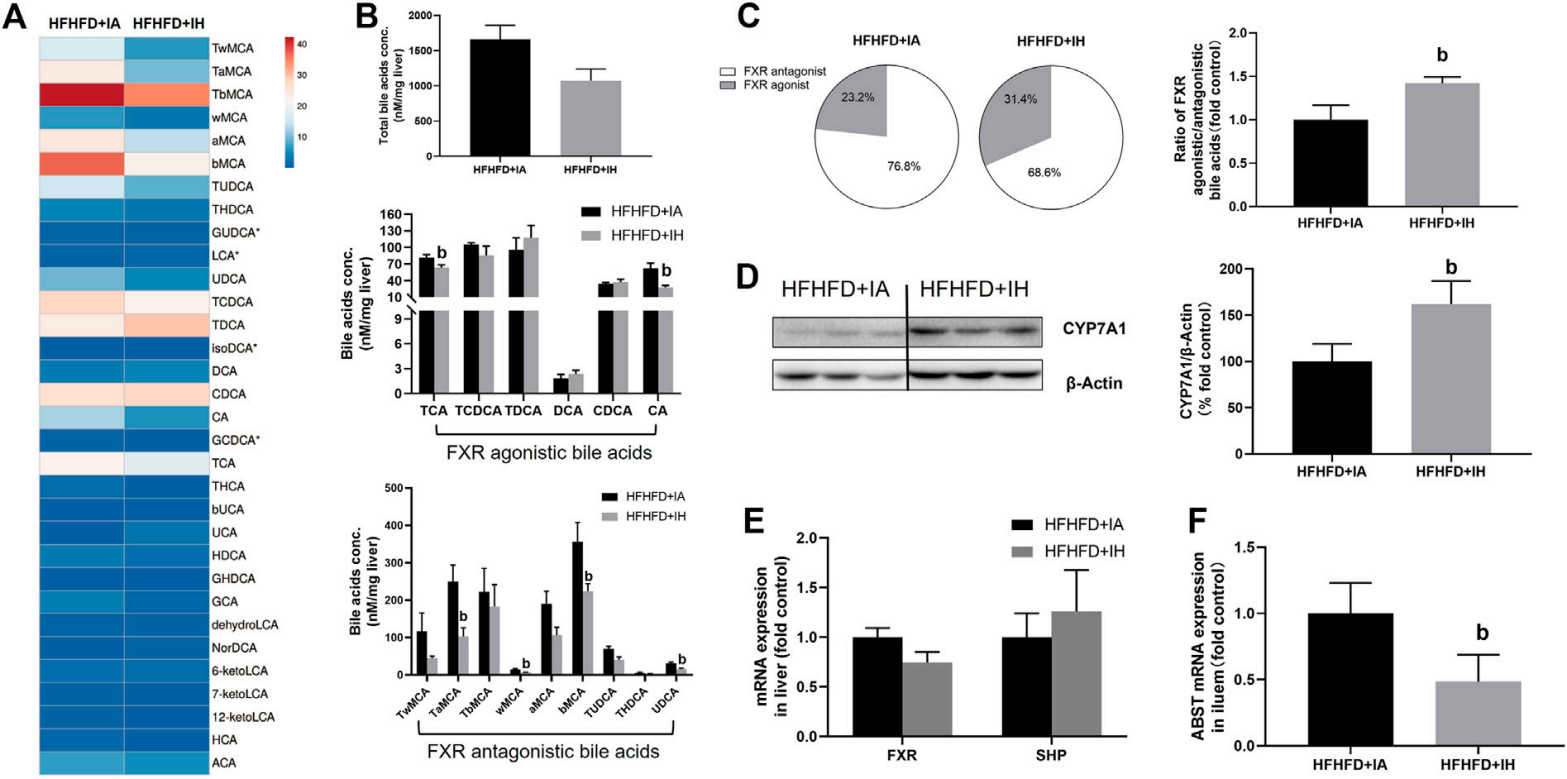
The effect of IH in bile acids metabolism in HFHFD-fed mice. **(A)** Heatmap comparing hepatic bile acids pool in HFHFD + IA versus HFHFD + IH group. **(B)** Total hepatic bile acids content and composition are shown in HFHFD-fed mice. **(C)** Pie charts for hepatic FXR agonist (grey)/antagonist (white) bile acids content are exhibited. The ratio of FXR agonistic to antagonistic bile acids content is shown in the right panel. **(D)** Representative western blots and densitometric analysis for CYP7A1 protein are shown. **(E)** Hepatic mRNA expressions of gene involved in bile acid signaling are shown. **(F)** Ileal ASBT gene level for bile acids transport is displayed. b, *p* < 0.05 compared to the absence of IH. Samples size per group n = 4–6

**FIGURE 9:**
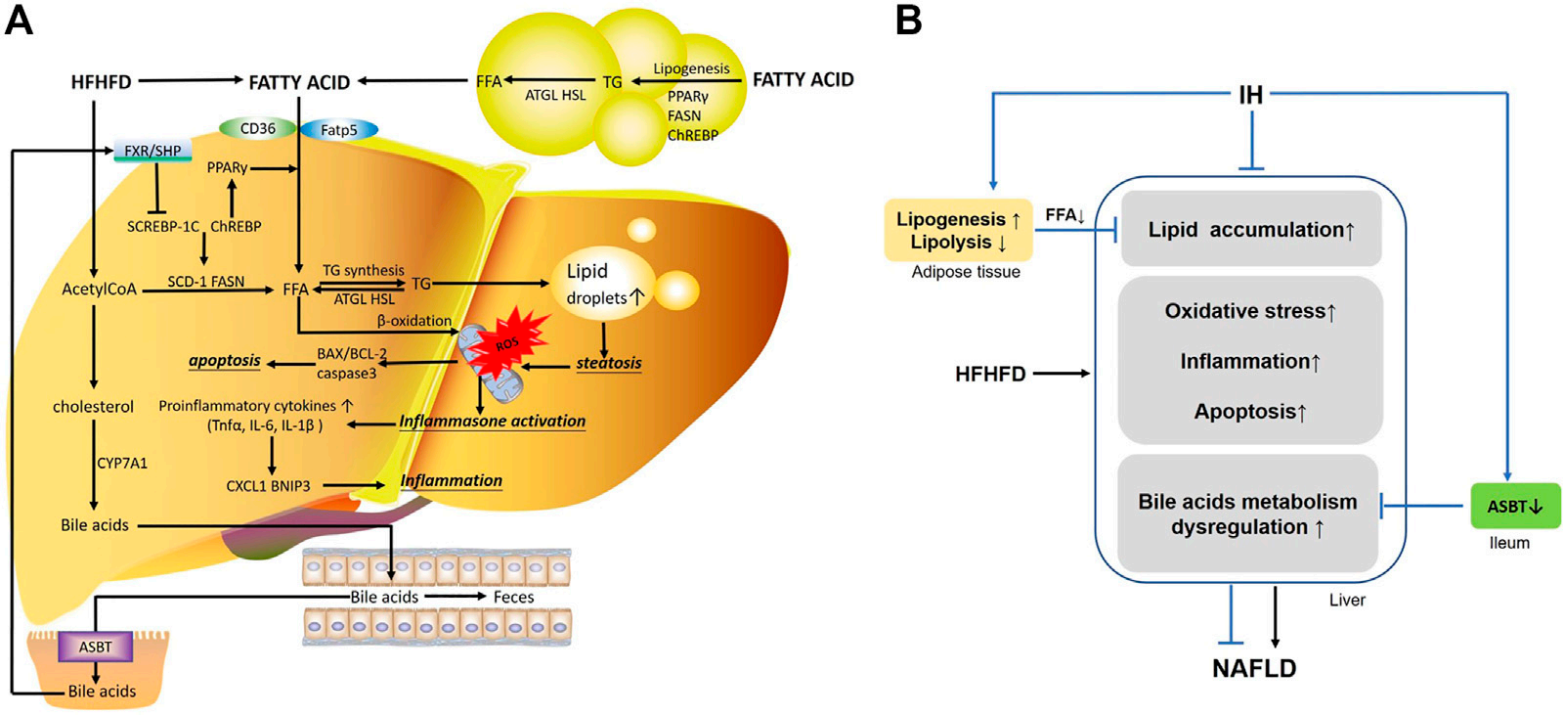
The main mechanisms involved in the interaction of IH and HFHFD. **(A)** FFA in the liver are from different sources, including FFA uptake from diet or lipolysis in adipose tissue *via* CD16 or Fatp5, and *de novo* lipogenesis stimulated by Srebp-1c, Chrebp and PPAR-γ. HFHFD-induced FFA and acetylCoA flux are shunted to lipid synthesis (TG) rather than β-oxidation, leading to steatosis. Overwhelmed lipids impair mitochondrial function, promote ROS production and cause oxidative stress. Meanwhile, inflammasomes are activated and increasing pro-inflammatory cytokines or chemokines (e.g., TNFα, IL-6, IL-1β, BNIP3 and CXCL1) are released. These stressful conditions influence the pro-apoptotic and anti-apoptotic factor (e.g., BAX, BCL-2) and trigger apoptosis, resulting in exacerbated liver injury. Additionally, part of acetylCoA is converted into cholesterol, which is the substrate for bile acids synthesis by CYP7A1. Hepatic bile acids are excreted into the gut lumen, most of which are reabsorbed by ileal ASBT and transported back to the liver *via* the portal circulation. Bile acid activates FXR/SHP axis and functions as a regulator in cellular physiological procedures such as lipid metabolism, playing an important role in liver function. **(B)** IH exposure protects liver from lipid accumulation, oxidative stress, inflammation and apoptosis in HFHFD mice. The preventive effects of IH liver injury are also achieved by limited uptake of FFAs mobilized from adipocyte tissue, and interruption of the enterohepatic bile acids circulation by ASBT inhibition.

Bile acid serves critical physiological functions in regulating whole-body metabolism especially lipid homeostasis in the liver. The heatmap of liver bile acid profiles showed that IH exposure with HFHFD decreased some of the differential bile acids compared to that of HFHFD + IA mice (Figure 8A), but the total bile acids content from the sum of individual bile acid species in HFHFD + IH was not statistically different from that in the HFHFD + IA liver (Figure 8B). Nuclear FXR is identified as a ligand-activated transcription factor and biological sensor for metabolic regulation. FXR directly activates SHP and FXR/SHP axis plays a role in modulating lipid homeostasis (Watanabe et al., 2004; Kim et al., 2017). However, neither FXR nor SHP mRNA expression was different between HFHFD + IA and HFHFD + IH mice (Figure 8E). Bile acids such as TCA, TCDCA, TDCA, DCA, CDCA, and CA, act as the potent agonists of FXR, whereas TUDCA, THDCA, UDCA, TMCAs, and MCAs function as FXR antagonists (Tu et al., 2000; Wang et al., 2008; Feng et al., 2011; Kumari et al., 2020). Administration of IH altered the composition of bile acids pool in HFHFD, as shown by decreased concentration of TCA, CA, TaMCA, wMCA, bMCA and UDCA (Figure 8B). As a result, IH rose the percentage of FXR agonistic bile acids in total bile acids content from 23.2% to 31.4%. The ratio of FXR agonistic to FXR antagonistic bile acids species in HFHFD + IH performed approximately 1.5-fold of that in HFHFD + IA (Figure 8C), suggesting that IH yielded bile acids pool prone to be, in sum, more FXR agonistic. To determine the basis for the altered bile acids profile, the protein expression of CYP7A1, responsible for bile acid synthesis, and mRNA of ileal ASBT which is critical for bile acids absorption, were detected by western blot and qPCR, respectively. In the HFHFD mice, IH showed a statistically increased CYP7A1 expression in the liver and decreased ASBT gene level in the ileum. (Figure 9). These data implied us bile acids metabolism regulation was involved in the interaction of IH and HFHFD.

## Discussion

It is accepted that the “Two-hit hypothesis” is viewed as a mechanism by which liver diseases initiate and progress to more severe states. The primary risk factor, such as high calories diet, is modified by additional environmental factors. OSA-generated IH seems to be such an environmental factor that plays a pivotal role in the exacerbation of liver injury (Zhou et al., 2021). In this study, we exposed mice to IH for 15 weeks and fed them with ND or HFHFD concurrently. Even though the pathology score and transaminase level in IH-exposed mice of ND group showed no significant change compared to IA-exposed mice of ND group, the increased FFA level, neutrophil infiltration, and apoptosis in the IH + ND mice proved that IH potentially contributed to liver damage in the ND mice. Rey et al. demonstrated that IH increased FFA by upregulating hepatic CD36 expression (Rey et al., 2020). IH increased hypoxia inducible factor (HIF-1α), which activated multiple transcription factors (NF-Kb, TNFα, and cleaved caspase 3), resulting in apoptosis (da Rosa et al., 2012). It was reasonable to speculate that HFHFD addition exacerbated liver injury in the IH-exposed mice. However, the combination of the western diet and IH exposure does not always follow two-hit rules. Some studies had shown that IH was beneficial to the liver function of fat-fed mice (Maeda and Yoshida, 2016). Indeed, we found that IH improved steatosis, oxidative stress, inflammation and apoptosis in the liver of HFHFD mice, suggesting that IH prevented liver injury from HFHFD. And these data also revealed an opposite effect of IH on mice fed with ND or HFHFD, indicating that IH interacting with HFHFD played a different role in liver function. The relationships and putative pathways linking IH, HFHFD and liver function are elaborated in the discussion below.

In our experiments, increased mice body weight, exacerbated macrovesicular steatosis, and impaired lipid/carbohydrates homeostasis such as hyperlipidemia (FFA, TG) and hyperglycemia, were observed in HFHFD treated mice. IH exposure decreased body weight gain, attenuated liver steatosis and decreased hepatic lipid content but did no change in either glucose tolerance or insulin sensitivity (Figures 2, 4). It suggested that IH in our model affected lipid metabolism rather than carbohydrate homeostasis. TG is the main store of lipids in the hepatocytes. IH markedly decreased TG level was partially due to the reduced fat mobilization, as proved by inhibited HSL and AGTL. FFA in the liver is from plasmatic FFA, dietary FFA, and *de novo* lipogenesis, plasmatic FFA comes mainly from lipolysis in the adipose tissue (Machado and Cortez-Pinto, 2014). The balance between FFA oxidation and TG synthesis affected lipid homeostasis in the liver. IH increased CPT-1α and decreased Fatp5, Chrebp, Srebp-1c, Scd-1 and PPAR-γ in HFHFD, indicating that IH protected the liver from lipid accumulation by elevated FFA oxidation, inhibited uptake of FFA, and suppressed lipogenesis. Interestingly, the effect of IH in eWAT was different from that in the liver. Luciano F. Drager et al. showed that lipolysis in adipose was inhibited by IH (Drager et al., 2011). In our model, IH reinforced the expression of lipogenesis-associated factors, Chrebp and Fasn, and decreased ATGL expression, resulting in the exacerbated eWAT steatosis and diminished lipolysis in HFHFD eWAT (Figure 5), which also explained the reduced serum FFA level in the HFHFD + IH. Collectively, these data proved that IH reduced liver steatosis not only *via* lipid metabolism regulation in the liver *per se* but also attributed to the less uptake of FFA from inhibited lipolysis in adipose.

Increasing evidence demonstrates that steatosis is correlated with oxidative stress. Lipid is the major target of ROS and, in turn, the intracellular accumulation of lipid contributes to oxidative stress (Poli, 2000). In previous work of our lab, we demonstrated that extra dietary fat caused mitochondrial dysfunction and resulted in exacerbated lipid peroxidation (Lang et al., 2018; Chen et al., 2019). Likewise, HFHFD aggravated the 4-HNE level as we expected, and this was blunted by IH treatment (Figure 4). We assumed that IH reduced oxidative stress in HFHFD might be due to: 1) Decreased lipid accumulation: As we mentioned above, IH inhibited lipid storage in the liver of HFHFD by manipulating metabolic homeostasis, and that might partly explain the effect of IH on lipid peroxide; 2) Antioxidants production: Maeda et al. reported that IH exposure increased hepatic expression of antioxidant enzymes and limited hepatic pathogenesis (Maeda and Yoshida, 2016). Consistently, we found that IH exposure upregulated nuclear Nrf2 expression in the HFHFD group. Increased Nrf2 translocation into the nucleus binds to ARE (antioxidant response element) and activated the transcription of downstream target genes such as SOD and CAT (Figure 4), and plays a role in alleviating oxidative stress (Ma et al., 2015); 3) Restored mitochondrial function: It is well known that mitochondrial dysfunction caused by a fat diet evokes the production of ROS and results in oxidative stress. Wojciech Trzepizur et al. had shown that impaired mitochondrial complex I and IV activities in high-fat diet-fed mice were restored by IH, contributing to preserved mitochondrial oxidative-phosphorylation (Trzepizur et al., 2015). Moreover, the role of IH in preventing mitochondria Ca^2+^ overload as well as cell death was elucidated by Chang’s work (Chang et al., 2019).

Inflammation and apoptosis were considered the critical mechanisms for NAFLD progression (Bessone et al., 2019). The inflammatory process is associated with inflammasome activation and pro-inflammatory cytokine production (Kim et al., 2020). Inflammasome assembly is triggered by ROS and participates in the inflammatory mediator secretion, leading to cell damage (Khan et al., 2017). Pro-inflammatory cytokines including IL-6 and TNFα were significantly decreased by IH treatment in the HFHFD mice liver. These factors are responsible for chemokine expression through the NF-kB pathway (Segerer et al., 2000; Galliera et al., 2008; Sanz et al., 2010). We found that CXCL1 was decreased in the liver of HFHFD-fed mice under IH conditions, while MCP-1 expression was not significantly changed by IH (Figure 6). That finding suggested that IH might regulate host immune response *via* recruiting neutrophils rather than monocyte or macrophages. Besides, we measured other factors for a further investigation of mechanisms in inflammation. HIF-1a is activated during hypoxia and implicated in inflammatory response (Dai et al., 2009). BNIP3 is the downstream target gene of HIF-1a and contributes to inflammation (Zhu et al., 2019; Irazoki et al., 2022). IH exhibited a decreased BNIP3 level in the HFHFD liver compared to HFHFD + IA. However, in the HFHFD mice, there was no detectable difference in HIF-1a expression between IA and IH exposure. That indicated the decreased BNIP3 might result from the reduction of TNFα by IH (Kim et al., 2011). Enlarged adipose tissue in obesity enhances the production of inflammatory chemokines and cytokines and decreased the production of beneficial ones, contributing to NAFLD progression (Rotter et al., 2003). We found IH did not alter the expression of anti- (adiponectin) and pro-inflammatory adipokine (IL-6, TNFα, ANGPTL2) (Fasshauer and Blüher, 2015) (Figure 6), as well as the macrophage infiltration marker F4/80 in the eWAT of HFHFD (supplemental material), suggesting that HFHFD induced inflammatory response in adipose was not markedly influenced by IH.

The pro-inflammatory condition in liver activates apoptotic pathway *via* the external activation of plasma membrane cytokine receptors to their cytokine ligands such as TNFR (by TNFα), activation of these receptors induces caspase3 and leads to apoptosis (Waring and Müllbacher, 1999). The excess lipid accumulation also triggers apoptosis by the intrinsic mitochondrial pathway. It is initiated by the activation of pro-apoptotic members (e.g., BAX), which is antagonized by anti-apoptotic members (e.g.,BCL-2) (Siddiqui et al., 2015). An alleviated TUNEL staining in the HFHFD liver was observed under IH condition, indicating that IH attenuated liver apoptosis in dietary fat or fructose-fed mice, as proved by reduced caspase3 cleavage and the ratio of BAX to BCL-2 (Figure 7). This result was also consistent with Dong et al. (2003)‘s work.

As we discussed so far, the mechanisms of liver injury including oxidative stress, inflammation and apoptosis, are partly related to lipotoxicity (e.g., FFA and TG). Our data showed that HFHFD promoted cholesterol levels in the liver and serum were decreased by IH exposure addition, implying that cholesterol and its metabolites were involved in the IH protection of liver function (Figure 4). Bile acids are synthesized from cholesterol in the liver *via* CYP7A1 (Evangelakos et al., 2021). IH enhanced CYP7A1 expression (Figure 8) and reduced liver cholesterol levels in the HFHFD (Figure 4). However, the total bile acids pool in the HFHFD + IH showed no significant difference from that of HFHFD + IA. We assumed it was due to the inhibited reabsorption from the ileum. ASBT is located in the intestine and controls bile acids uptake from the lumen and conveys them through the portal vein to the liver (Dawson and Karpen, 2015). As we expected, IH exposure inhibited the ASBT expression in the HFHFD mice ileum (Figure 8). The increased bile acids synthesis and decreased reabsorption caused no obvious change in total bile acids content between HFHFD + IA and HFHFD + IH mice. FXR/SHP plays a role in NAFLD protection by regulating lipid homeostasis and anti-inflammation (Fiorucci et al., 2004; Watanabe et al., 2004; Carr and Reid, 2015). In the HFHFD mice, IH did not affect FXR or SHP expression compared to IA exposure, but led to a marked shift in hepatic bile acid composition, with a reduction of FXR antagonistic species and an increase in FXR agonistic bile acids (Figure 8). Thus, more FXR was activated in response to this alteration, contributing to liver protection. Consistent with our findings, Rao et al. also reported that inhibition of ileal ASBT performed more FXR agonistic bile acids and protected against NAFLD in high-fat diet mice (Rao et al., 2016). Taken together, we thought IH prevented liver injury from HFHFD *via* interruption of the enterohepatic bile acids circulation and alteration of bile acids composition.

## Conclusion and future prospects

These data show that IH exposure prevents against HFHFD-induced liver injury. IH attenuated liver steatosis *via* enhanced lipid usage and inhibited lipid synthesis, that reduced lipid peroxidation, inflammation and apoptosis. Moreover, IH regulated bile acids signaling pathway and prevented liver injury progression. A limitation of the current work is that it does not build animal models at different time points. Recent studies indicated that IH performed a dimorphic role in liver function. IH exposure showed severer liver injury in the 6-week model of NAFLD (Feng et al., 2011). Wojciech Trzepizur et al. revealed that 14-days IH exposure prior to sacrifice restored mitochondrial function and limited lipid accumulation in 8-week mice model of a high-fat diet (Trzepizur et al., 2015). It is yet to be determined if IH duration is a factor that impacts the underlying mechanisms and liver function in HFHFD. Future studies should focus on these aspects to fill the gaps in our knowledge.

## Data Availability

The raw data supporting the conclusion of this article will be made available by the authors, without undue reservation.

## Glossary

NAFLD: Non-alcoholic fatty liver disease
OSA: Obstructive sleep apnea
IH: Intermittent hypoxia
IA: Intermittent room air
ND: Normal diet
HFHFD: High-fat: high-fructose diet
GTT: Glucose tolerance test
ITT: Insulin tolerance test
ALT: Alanine aminotransferase
AST: Aspartate aminotransferase
TG: Triglyceride
FFA: Free fatty acid
4-HNE: 4-Hydroxynonenal
H&E: Hematoxylin and eosin
ORO: Oil Red O
CAE: Chloroacetate esterase
TUNEL: Terminal deoxynucleotidyl transferase–mediated deoxyuridine triphosphate nick-end labeling
eWAT: Epididymal white adipose tissues
HSL: Hormone-sensitive lipase
ATGL: Adipose triglyceride lipase
Cd36: Fatty acid translocase
Fatp5: Fatty acid transport protein 5
CPT-1α: Carnitine palmitoyl transferase-1α
Fasn: Fatty acid synthase
Chrebp: Carbohydrate response element-binding protein
Srebp-1c: Sterol regulatory element-binding protein-1c
Scd-1: Stearoyl-CoA desaturase-1
PPAR-γ: Peroxisome proliferator-activated receptor γ
SOD: Superoxide dismutase
CAT: Catalase
Nrf2: Nuclear erythroid 2-related factor 2
CXCL1: C-X-C Motif Chemokine Ligand 1
MCP-1: Monocyte chemotactic protein-1
TNFα: Tumor necrosis factor-α
IL-6,1β: Interleukin-6, 1β
BNIP3: BCL2 Interacting Protein 3
HIF-1a: Hypoxia-inducible factor -1a
ANGPTL2: Angiopoietin-related protein 2
BAX: Bcl-2-associated X protein
BCL-2: B cell lymphoma-2
ROS: Radical oxygen species
FXR: Farnesoid X receptor
SHP: Small heterodimer partner
ASBT: Apical sodium-dependent bile salt transporter
CYP7A1: Cytochrome P450 family 7 subfamily A member 1
TCA: Taurocholic acid Sodium Salt
TCDCA: Taurochenodeoxycholic acid
TDCA: Taurodeoxycholic acid sodium salt
DCA: Deoxycholic acid
CDCA: Chenodeoxycholic acid
CA: Cholic acid
TUDCA: Tauroursodeoxycholic acid sodium salt
THDCA: Taurohyodeoxycholic acid sodium salt
UDCA: Ursodeoxycholic acid
TMCAs: Tauro-muricholic acid sodium salt
MCAs: Muricholic acid
