# Supplementary material for: Improvement of obesity-induced fatty liver disease by intermittent hypoxia exposure in a murine model
Source: Front Pharmacol. 2023 Feb 15;14:1097641. doi: 10.3389/fphar.2023.1097641 (PMC9974667; doi:10.3389/fphar.2023.1097641)
Supplement: Supplementary file 1 [file Table1.DOCX]

Supplemental material and method

Sirius Red staining

Liver slides were stained for Sirius Red to evaluate liver fibrosis using a commercial kit (Chondrex. lnc) as described by an instructional protocol.

Real-time qPCR

Total RNA was used for reverse transcription with the cDNA cycle kit (Invitrogen). Collagen I (5’-CCTCAGGGTATTGCTGGACAAC, 3’- CAGAAGGACCTTGTTTGCCAGG) and III (5’- GACCAAAAGGTGATGCTGGACAG, 3’- CAAGACCTCGTGCTCCAGTTAG) primers were purchased from OriGene. Real-time PCR was performed on the ABI 7300 fast real-time PCR system (Applied Biosystems) using SYBR green PCR Master Mixture (Applied Biosystems). The 2^-△△CT^ method was used to determine fold differences between the target genes and an endogenous reference (GAPDH).

Western Blot

Whole protein was extracted from frozen eWAT. Protein concentration was determined using a Pierce BCA Protein Assay kit (Thermo Fisher Scientific, MA). Primary antibodies (1:1000) against F4/80 was from Santa Cruze Biotechnology (USA). Densitometric analysis was performed using UN-SAN-IT Gel (Silk Scientific, Orem, UT) software.

Supplemental Figure.

A: Representative photomicrographs of Sirius Red staining for all the groups were performed. B: The mRNA expression of liver fibrogenic gene Collagen I and Collagen III were performed in HFHFD+IA and HFHFD+IH. C：Representative western blots and densitometric analysis for whole eWAT F4/80 protein is shown. Samples size per group n=4–6.
