# Supplementary figures and images for: Improvement of obesity-induced fatty liver disease by intermittent hypoxia exposure in a murine model
Source: Front Pharmacol. 2023 Feb 15;14:1097641. doi: 10.3389/fphar.2023.1097641 (PMC9974667; doi:10.3389/fphar.2023.1097641)

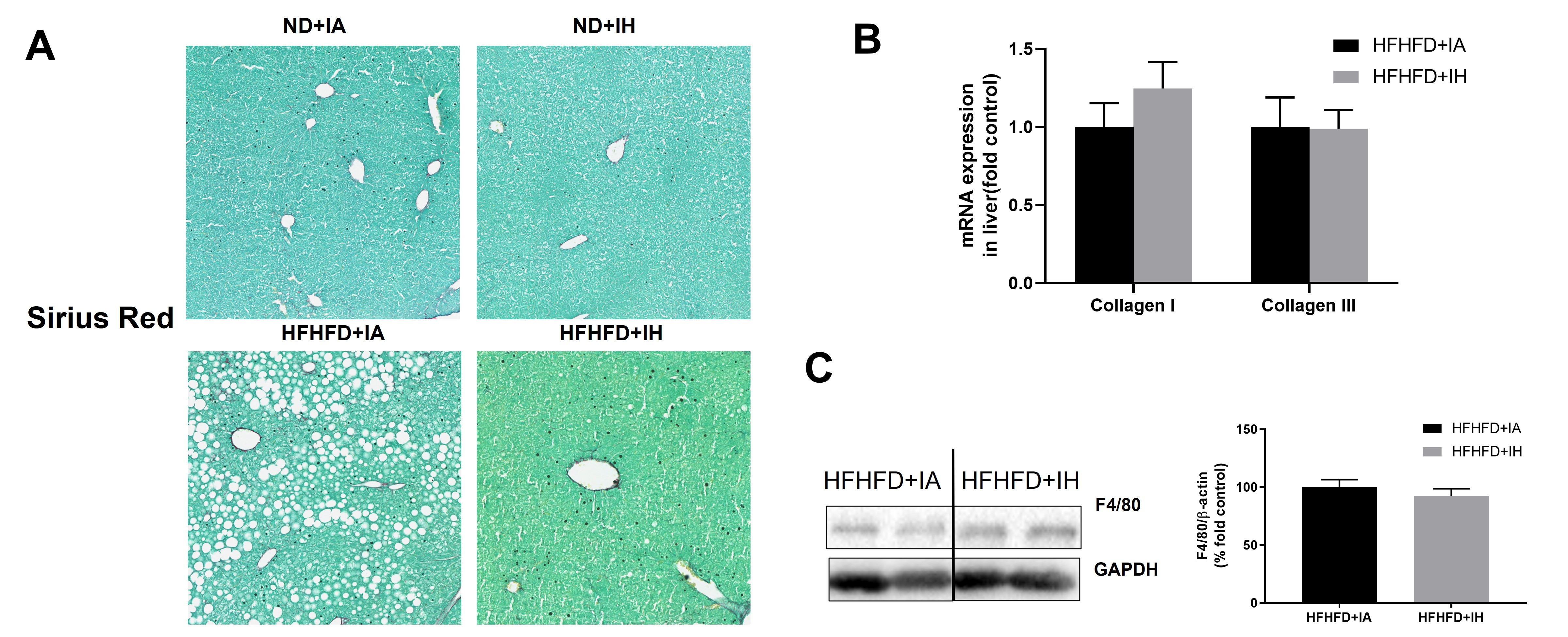

Supplement: Supplementary file 2 [file Image1.JPEG]
