# Supplementary material for: Improvement of obesity-induced fatty liver disease by intermittent hypoxia exposure in a murine model
Source: Front Pharmacol. 2023 Feb 15;14:1097641. doi: 10.3389/fphar.2023.1097641 (PMC9974667; doi:10.3389/fphar.2023.1097641)
Supplement: Supplementary file 3 [file DataSheet1.PDF]

Product#	D09100308L		D12450J	
	Primex			
	gm%	kcal%	gm%	kcal%
Protein	22.5	20	19.2	20
Carbohydrate	44.9	40	67.3	70
Fat	19.9	40	4.3	10
Total		100		100
kcal/gm	4.49		3.85	
**Ingredient**	**gm**	**kcal**	**gm**	**kcal**
Casein	200	800	200	800
L-Cystine	3	12	3	12
corn Starch	0	0	506.2	2024.8
Maltodextrin 10	100	400	125	500
Fructose	200	800	0	0
Sucrose	96	384	68.8	275.2
Cellulose	50	0	50	0
Soybean Oil	25	225	25	225
Lard	20	180	20	180
Primex Shortening	135	1215	0	0
Palm Oil	0	0	0	0
Mineral Mix S10026	10	0	10	0
DiCalcium Phosphate	13	0	13	0
Calcium Carbonate	5.5	0	5.5	0
Potassium Citrate, 1 H2O	16.5	0	16.5	0
Vitamin Mix V10001	10	40	10	40
Choline Bitartrate	2	0	2	0
Cholesterol	18	0	0	0
FD&C Yellow Dye #5	0.05	0	0.04	0
FD&C Red Dye #40	0	0	0	0
FD&C Blue Dye #1	0	0	0.01	0
Total	904.05	4056	1055.05	4057
